# Artificial intelligence-based analyses of varus leg alignment and after high tibial osteotomy show high accuracy and reproducibility

**DOI:** 10.1007/s00167-023-07644-0

**Published:** 2023-11-17

**Authors:** Christoph Stotter, Thomas Klestil, Kenneth Chen, Allan Hummer, Christoph Salzlechner, Peter Angele, Stefan Nehrer

**Affiliations:** 1 Department for Orthopedics and Traumatology, Landesklinikum Baden-Mödling, 2340 Mödling, Austria; 2 Department for Health Sciences, Medicine and Research, University for Continuing Education Krems, 3500 Krems, Austria; 3 ImageBiopsy Lab, 1140 Vienna, Austria; 4 Sporthopaedicum Regensburg, 93053 Regensburg, Germany; 5 Clinic for Trauma and Reconstructive Surgery, University Medical Center Regensburg, 93042 Regensburg, Germany

**Keywords:** Deep learning, AI, Deformity analysis, HTO

## Abstract

**Purpose:**

The aim of this study was to investigate the performance of an artificial intelligence (AI)-based software for fully automated analysis of leg alignment pre- and postoperatively after high tibial osteotomy (HTO) on long-leg radiographs (LLRs).

**Methods:**

Long-leg radiographs of 95 patients with varus malalignment that underwent medial open-wedge HTO were analyzed pre- and postoperatively. Three investigators and an AI software using deep learning algorithms (LAMA™, ImageBiopsy Lab, Vienna, Austria) evaluated the hip–knee–ankle angle (HKA), mechanical axis deviation (MAD), joint line convergence angle (JLCA), medial proximal tibial angle (MPTA), and mechanical lateral distal femoral angle (mLDFA). All measurements were performed twice and the performance of the AI software was compared with individual human readers using a Bayesian mixed model. In addition, the inter-observer intraclass correlation coefficient (ICC) for inter-observer reliability was evaluated by comparing measurements from manual readers. The intra-reader variability for manual measurements and the AI-based software was evaluated using the intra-observer ICC.

**Results:**

Initial varus malalignment was corrected to slight valgus alignment after HTO. Measured by the AI algorithm and manually HKA (5.36° ± 3.03° and 5.47° ± 2.90° to − 0.70 ± 2.34 and − 0.54 ± 2.31), MAD (19.38 mm ± 11.39 mm and 20.17 mm ± 10.99 mm to − 2.68 ± 8.75 and − 2.10 ± 8.61) and MPTA (86.29° ± 2.42° and 86.08° ± 2.34° to 91.6 ± 3.0 and 91.81 ± 2.54) changed significantly from pre- to postoperative, while JLCA and mLDFA were not altered. The fully automated AI-based analyses showed no significant differences for all measurements compared with manual reads neither in native preoperative radiographs nor postoperatively after HTO. Mean absolute differences between the AI-based software and mean manual observer measurements were 0.5° or less for all measurements. Inter-observer ICCs for manual measurements were good to excellent for all measurements, except for JLCA, which showed moderate inter-observer ICCs. Intra-observer ICCs for manual measurements were excellent for all measurements, except for JLCA and for MPTA postoperatively. For the AI-aided analyses, repeated measurements showed entirely consistent results for all measurements with an intra-observer ICC of 1.0.

**Conclusions:**

The AI-based software can provide fully automated analyses of native long-leg radiographs in patients with varus malalignment and after HTO with great accuracy and reproducibility and could support clinical workflows.

**Level of evidence:**

Diagnostic study, Level III.

**Supplementary Information:**

The online version contains supplementary material available at 10.1007/s00167-023-07644-0.

## Introduction

Indications for high tibial osteotomy (HTO) include medial knee osteoarthritis (OA) in patients with a bony varus deformity [7] and patients with cartilage repair surgery and varus malalignment [2, 14]. A thorough analysis of the leg alignment is mandatory for detection of malalignment and for locating the bony deformity, as HTO is indicated only in patients with the varus malalignment located in the proximal tibia [9]. Preoperative measurements in the coronal plane are usually performed on standing anteroposterior long-leg radiographs [16]. Traditionally, these measurements are performed manually using medical imaging software. However, subjective landmark setting leads to high intra- and inter-observer variability, poor reproducibility [3, 10, 20] and measurements depend on the experience of the observers [25].

Artificial intelligence and machine learning are increasingly applied in musculoskeletal imaging [5, 15]. Various machine learning models have been developed [18] and demonstrated that automated analysis using AI and deep learning algorithms can enhance measurements and increase reproducibility [24]. Classical machine learning methods can be labeled as either supervised or unsupervised methods [8, 17]. Supervised machine learning methods predict health outcomes based on labeled data [17], while unsupervised machine learning methods identify patterns in unlabeled data sets and can identify new risk factors [8]. The American College of Radiology Data Science Institute has recognized leg length measurement in radiographs as an AI use case. Studies for angle measurements on native long-leg radiographs with neutral alignment and postoperatively after total knee arthroplasty (TKA) showed good results compared with manual readers [12, 21, 23].

However, no study has investigated the performance of artificial intelligence enhanced analysis of varus malalignment or after high tibial osteotomy. In the clinical environment, the utilization of an AI-based software has the potential to save resources, provide fast delivery of precise results and support clinical decision-making.

The aim of this study was to apply an AI-based software on long-leg radiographs in patients with symptomatic varus malalignment pre- and postoperatively after high tibial osteotomy. The software based on deep learning algorithms provides a fully automated analysis of leg alignment and bony deformity. It was hypothesized that the AI-based software can accurately analyze leg alignment pre- and postoperatively after HTO. Second, it was hypothesized that the software performs equally compared with manual readers and that inclusion of the software as an individual reader does not alter the accuracy.

## Materials and methods

Patients that received a medial open-wedge high tibial osteotomy between May 2019 to December 2020 at our institutions were identified. Inclusion criteria were patients aged 18 years or older, who were treated with an HTO and had at least one weight-bearing long-leg radiograph preoperatively and one at a minimum of 6 weeks postoperatively. Exclusion criteria were previous surgery with detectable implants in the proximal tibia, radiographs with artifacts, poor image quality, incorrect positioning and cropping errors. Image quality was assessed again by each reader before starting the annotation process. The assessment included checks for incorrect image cropping, clear visibility of bone contours, excessive tilt and correct rotation of the lower limb. Correct rotation was evaluated by a combination of patella orientation, coverage of the fibular head, and configuration of the femoral notch. After a power analysis (see statistical methods) 95 patients with 190 radiographs were included in this study to guarantee sufficient sample size. Individual informed consent was waived by the local ethics committee due to the retrospective study design and anonymization of the data. All radiographs were acquired with the same device (DigitalDiagnost, Philips).

### Manual measurements

Manual measurements were carried out by three investigators independently. Two of them are orthopedic surgeons and one of them is a radiologist, all with a minimum of 5 year experience in musculoskeletal imaging. The annotations were obtained using mediCAD® (Knee 2D module v6.0, mediCAD Hectec GmbH, Altdorf/Landshut, Germany) according to the user’s manual workflow. Each reader was blinded to the AI results, worked independently and annotated each image twice in the same order. The measurements were exported from mediCAD® and collected in a Microsoft Excel sheet.

### Automated measurements using AI software

The AI-based software used in this study was the LAMA™ (Leg Angle Measurement Assistant) software (version 1.04.15, CE version, ImageBiopsy Lab, Vienna, Austria). It was trained on over 15,000 radiographs from the OAI (Osteoarthritis Initiative study; US six-site multi-center), MOST (Multicenter Osteoarthritis Study, US two-site multi-center), CHECK (Cohort Hip and Cohort Knee study; Netherland single center) studies as well as five sites in Austria [19]. LAMA™ uses deep learning algorithms and multiple U-Net-based convolutional neural networks. The software automatically annotates the original DICOM (Digital Imaging and Communications in Medicine) images and analyzes long-leg radiographs by detecting and localizing anatomically relevant landmarks on the femur and tibia relevant for the required measurements. In addition, if present, a calibration ball is identified and the resulting magnification factor for length measurements is applied. The AI follows the established radiological workflow: measurement of anatomical distances and angles, detection of disease morphologies and provides standardized reporting for all measurements. LAMA™ performs a consensus assessment for each radiograph. Every detection step is performed by three AI models that then vote for the appropriate result. The software consists of multiple convolutional deep neural networks (CNNs) which operate on either all or part of the input images and perform segmentation, landmarking and detection tasks. A detailed description of the calculation logic, the training model and the CNNs is provided in the supplement material of [23].

### Measurements

**Fig. 1:**
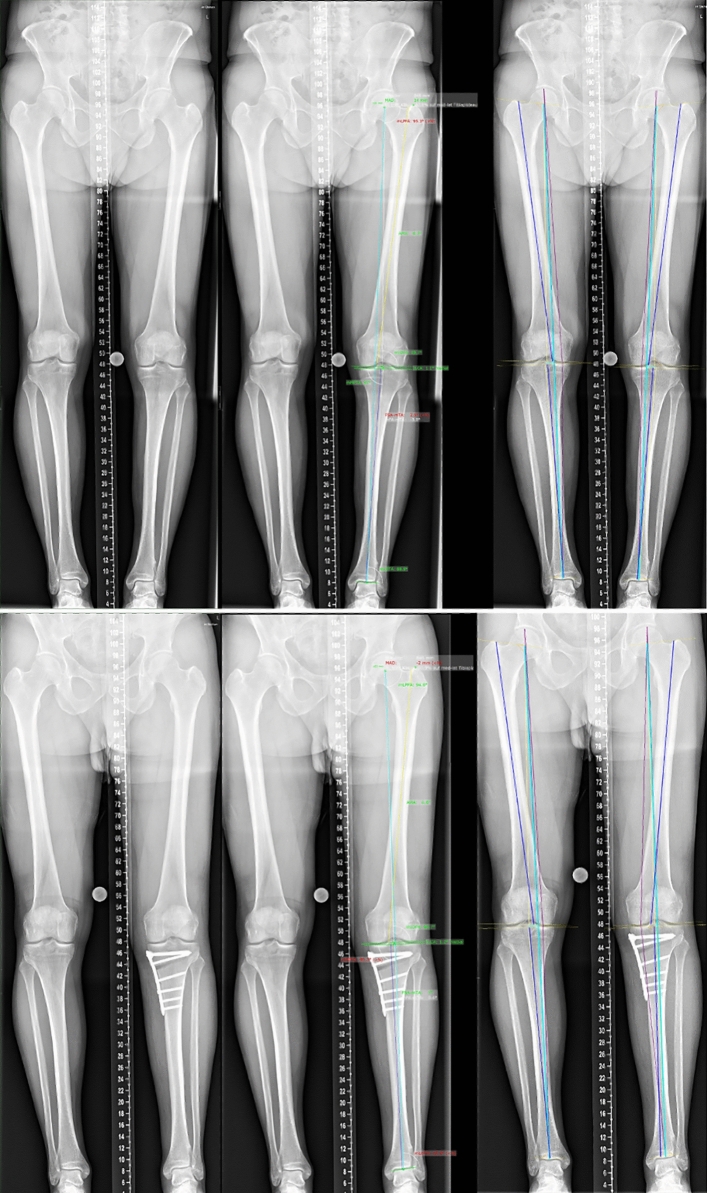
Representative images of weight-bearing long-leg radiographs of a patient with mild varus malalignment (top) and after medial opening wedge osteotomy (bottom). The figure shows native X-rays (left), manual measurements using mediCAD® (center), and fully automated analysis performed by an AI software (LAMA™); right). Measurements include mechanical axis deviation (MAD), hip–knee–ankle angle (HKA), joint line convergence angle (JLCA), mechanical lateral distal femoral angle (mLDFA), and medial proximal tibial angle (MPTA)

The measurements for manual and AI-aided analyses that were further evaluated included mechanical axis deviation (MAD), hip–knee–ankle angle (HKA), joint line convergence angle (JLCA), mechanical lateral distal femoral angle (mLDFA), and medial proximal tibial angle (MPTA) (see Fig. 1). Furthermore, standardized analyses included determination of full leg length, femur length, tibia length, anatomical–mechanical axis angle (AMA), mechanical lateral proximal femoral angle (mLPFA), and mechanical lateral distal tibial angle (mLDTA). Estimation of the magnification factor was performed using a 25 mm-calibration ball or ruler.

The study received approval from the Ethics Committee of Lower Austria, Austria (GS1-EK-3/171-2020).

### Statistical methods

We used a Bayesian approach in our analysis, which has several advantages over conventional frequentist methods. These advantages include ease of interpretation and the avoidance of issues related to null hypothesis significance testing. In our case, the Bayesian approach allowed us to compare the performance of individual human readers with the AI and account for the fact that there is no real ground truth available. For this purpose, we used the surface under the cumulative ranking (SUCRA). We ranked the readers and the AI-based on the absolute deviation from the median of ratings from all readers and the AI. All analyses were conducted in the R environment (version 4.2.1) using the tidyverse package for data wrangling and plot creation. The calculations were performed using Markov chain Monte Carlo via the brms package. We conducted a multi-level analysis taking into account that we had multiple measures per patient, with restrictive priors preventing negative values for the absolute deviation. The intercept was suppressed. To determine a sufficient sample size for this study we performed a power analysis using G*Power version 3.1.9.6. The effect size was based on reference values from previous studies and the level of significance and the power level were set at 0.05 and 0.90, respectfully. Based on these parameters, the minimal required sample to guarantee adequate power for statistical testing for the defined measurements was found to be 84. To guarantee sufficient statistical power, this number was increased and 95 patients with 190 radiographs were included in this study.

In addition, we conducted a sensitivity analysis, where we assessed whether the absolute deviation from the median is different in post-operative images depending on the rater. In this case, the intercept was included in the model. The intra- and inter-reader variability was evaluated using the Intraclass Correlation Coefficient (ICC). Values less than 0.50 were considered poor reliability, those between 0.50 and 0.75, as moderate reliability, those between 0.75 and 0.90 as good reliability, and those greater than 0.90 as excellent reliability.

## Results

A total of 190 radiographs from 95 patients were included in this study (age: 46.9 ± 7.6 years; 41 female, 54 male). Nine measurements in five radiographs were identified as outliers due to invalid landmark setting by the AI software and were excluded for all further analyses. These erroneous measurements were observed on two preoperative and three postoperative radiographs. The following plots exclude these outliers. Analyses and plots including erroneous measurements are provided in the supplement material for detailed error analysis.

**Fig. 2:**
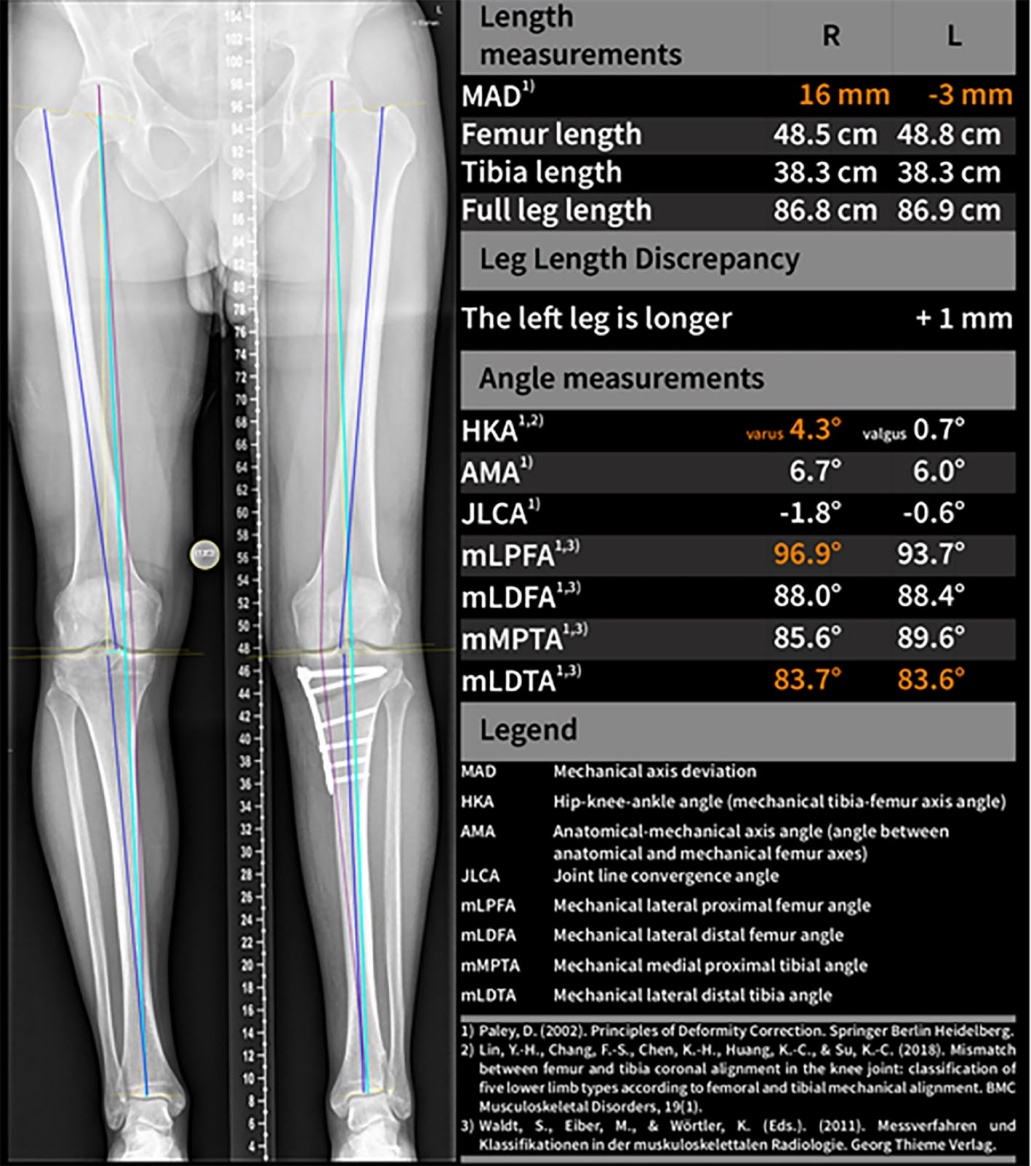
AI software (LAMA™) report of a bilateral weight-bearing long-leg radiograph after medial opening wedge osteotomy providing fully automated measurements

Naturally, HKA, MAD and MPTA changed significantly after HTO, while JLCA and mLDFA were not altered. Preoperative varus malalignment was corrected to slight valgus alignment. Table 1 summarizes pre- and postoperative measurements by the AI-based software and manual measurements. There were no significant differences between manual measurements and the fully automated AI-based analyses neither in preoperative radiographs nor after HTO (see supplement material) (Fig. 2).

**Table 1:** Pre- and postoperative radiographic measurements for LAMA™ and manual measurement

	LAMA™			Manual		
	Pre-op	Post-op	*p* value	Pre-op	Post-op	*p* value
HKA (°)	5.36 (± 3.03)	− 0.70 (± 2.34)	** < 0.0001**	5.47 (± 2.90)	− 0.54 (± 2.31)	** < 0.0001**
MAD (mm)	19.38 (± 11.39)	− 2.68 (± 8.75)	** < 0.0001**	20.17 (± 10.99)	− 2.10 (± 8.61)	** < 0.0001**
JLCA (°)	2.23 (± 2.0)	1.37 (± 2.86)	n.s	2.03 (± 1.84)	1.78 (± 1.83)	n.s
MPTA (°)	86.29 (± 2.42)	91.6 (± 3.0)	** < 0.0001**	86.08 (± 2.34)	91.81 (± 2.54)	** < 0.0001**
mLDFA (°)	89.42 (± 1.99)	89.48 (± 2.96)	n.s	89.52 (± 5.3)	89.49 (± 2.19)	n.s

All values are reported as mean ± standard deviation (SD). For MAD and HKA positive values (+) indicate varus, while negative values (−) indicate valgus

**Fig. 3:**
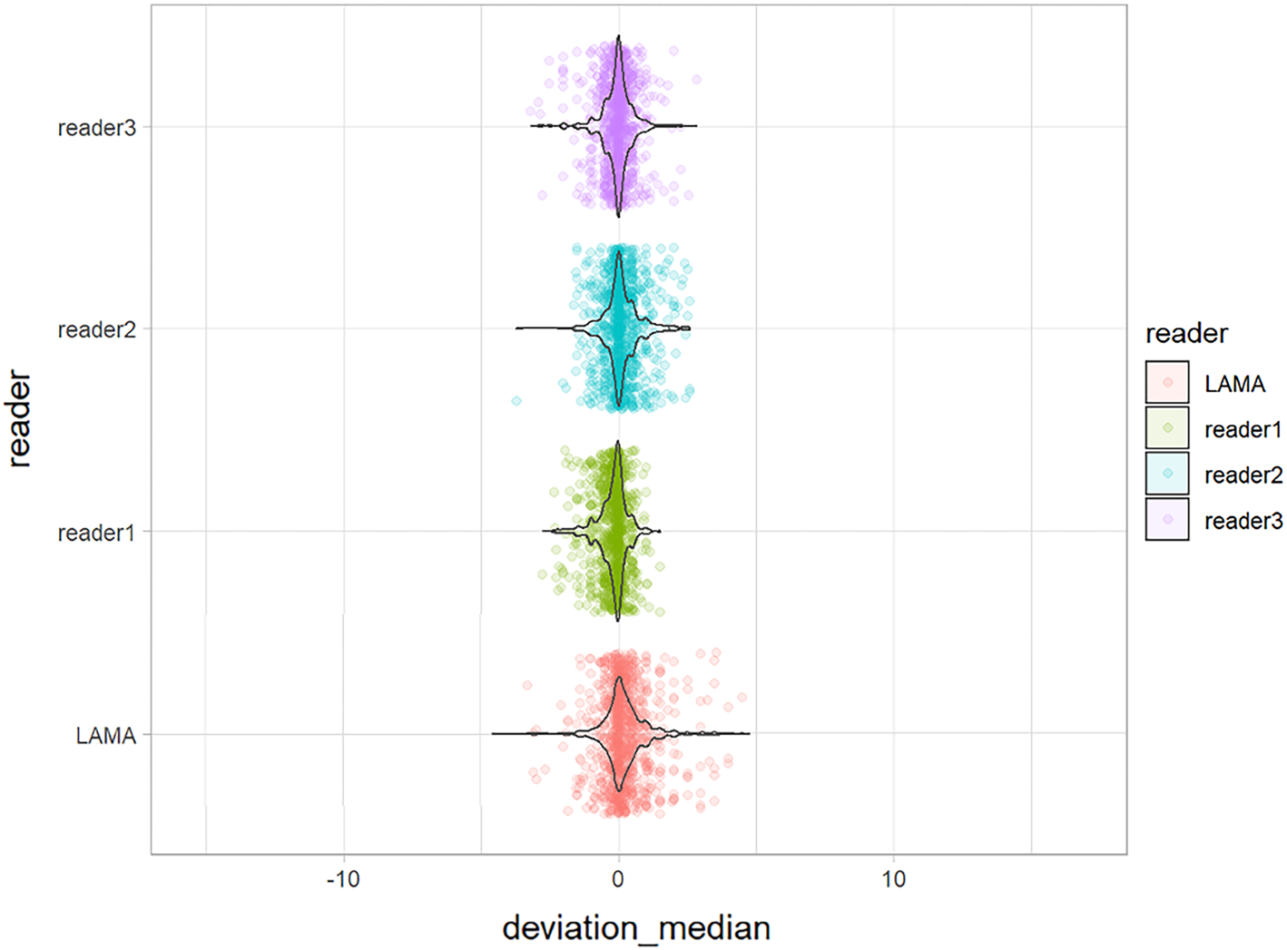
Deviation from the median for each individual observation of all readers and LAMA™

**Fig. 4:**
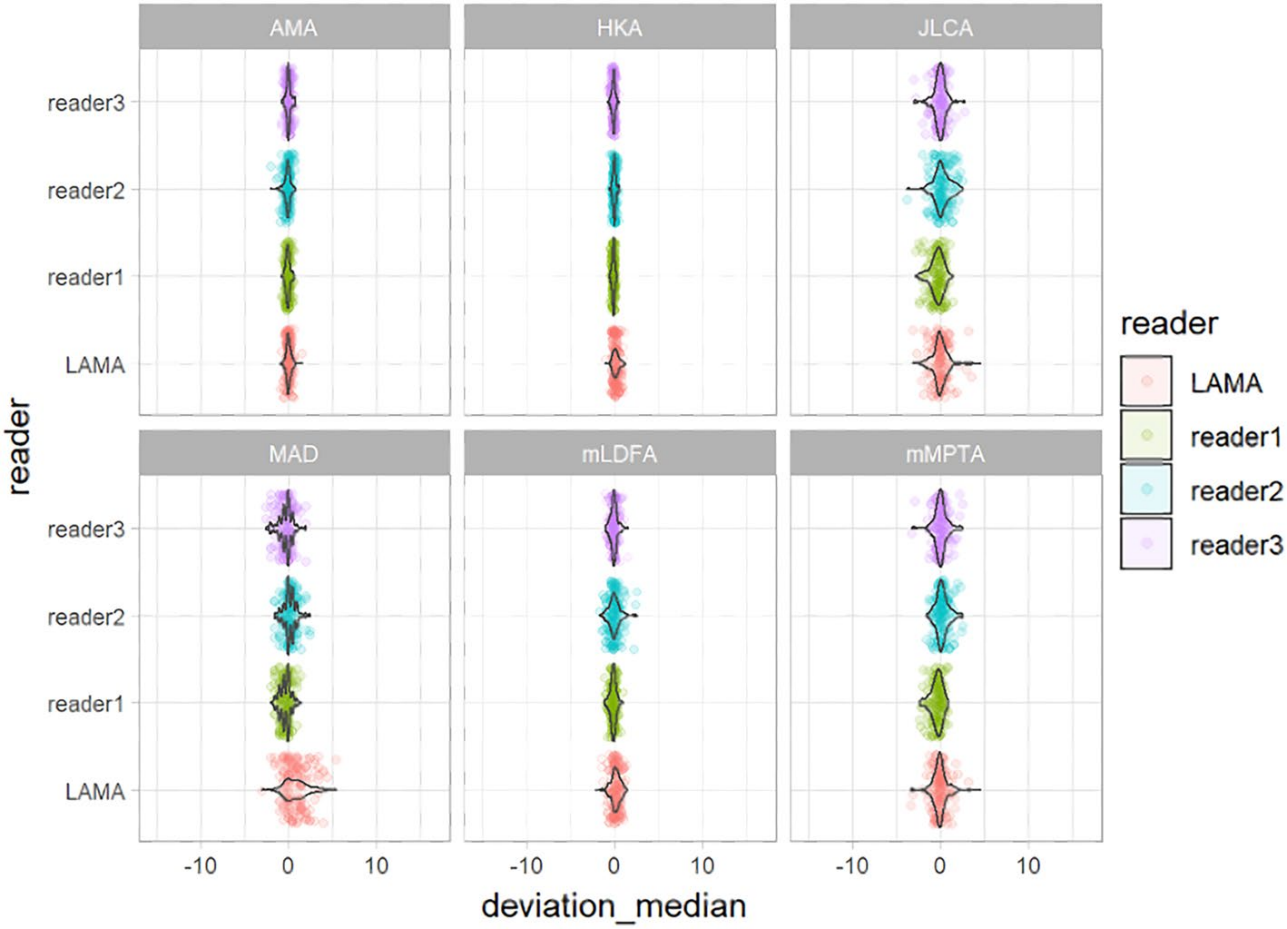
Deviations from the median for each individual observation for all measurements for reader 1–3 and LAMA™

**Fig. 5:**
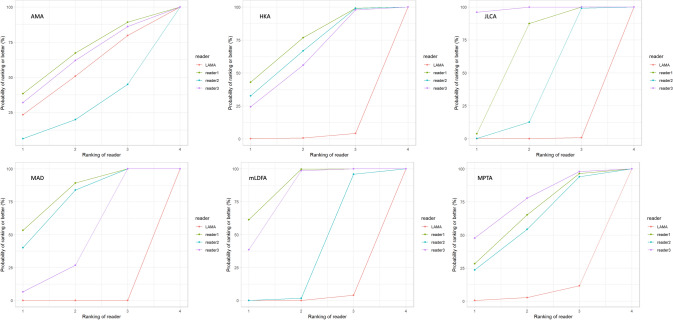
Surface under the cumulative ranking (SUCRA) plots for AMA, HKA, JLCA, MAD, mLDFA and MPTA. The plots indicate the probability that a reader ranks better than a certain rank (i.e., less absolute deviation from the median)

The deviations from the median for all observations are displayed in Fig. 3. The deviations from the median for each outcome measurement and all readers are displayed in Fig. 4. The corresponding absolute deviations are provided in the supplement material. The SUCRA plots show the probabilities that an individual reader ranks better (less absolute deviation from the median) than a certain rank (see Fig. 5). Except for the AMA, the AI software showed low probabilities to outperform manual readers. However, mean absolute differences between LAMA™ and mean manual observer measurements where below 0.5° and not statistically significant (*p* > 0.05) for all measurements (see supplement material).

Inter- and intra-observer ICCs for all measurements pre- and postoperatively are shown in Tables 2 and 3. Inter-observer ICCs for manual measurements were excellent for all measurements, except for JLCA, which showed moderate to good inter-observer ICCs, and MPTA postoperatively, which showed good inter-observer ICC. Intra-observer ICCs for manual measurements were excellent for all measurements, except for JLCA, which showed moderate to excellent ICCs and for MPTA postoperatively, which showed good to excellent ICCs. Repetitive analysis of pre- and postoperative radiographs using LAMA™ showed an entirely consistent reproducibility with an intra-observer ICC of 1.0, demonstrating consistent intra-rater variability.

**Table 2:** Inter-observer Intraclass Correlation Coefficients (ICCs) for manual radiographic measurements pre- and postoperatively (95% confidence interval)

	HKA	MAD	JLCA	MPTA	mLDFA
All	0.99 (0.99–1.00)	0.99 (0.99–1.00)	0.74 (0.68–0.80)	0.95 (0.93–0.96)	0.94 (0.93–0.96)
Pre-op	0.99 (0.99–1.00)	0.99 (0.99–1.00)	0.79 (0.71–0.86)	0.93 (0.89–0.95)	0.95 (0.92–0.97)
Post-op	0.98 (0.98–0.99)	0.98 (0.98–0.99)	0.71 (0.62–0.79)	0.87 (0.82–0.92)	0.94 (0.92–0.96)

**Table 3:** Intra-observer Intraclass Correlation Coefficients (ICCs) for repeated measurements of radiographic measurements for manual readers and LAMA™ (95% confidence interval)

	All				Pre-op				Post-op			
	Reader 1	Reader 2	Reader 3	LAMA™	Reader 1	Reader 2	Reader 3	LAMA™	Reader 1	Reader 2	Reader 3	LAMA™
HKA	0.99 (0.99–1.00)	0.99 (0.99–1.00)	0.99 (0.99–1.00)	1.00 (1.00–1.00)	1.00 (0.99–1.00)	1.00 (0.99–1.00)	1.00 (0.98–1.00)	1.00 (1.00–1.00)	1.00 (0.99–1.00)	0.98 (0.97–0.99)	0.98 (0.97–0.99)	1.00 (1.00–1.00)
MAD	0.99 (0.99–1.00)	0.99 (0.99–1.00)	0.99 (0.99–1.00)	1.00 (1.00–1.00)	1.00 (0.99–1.00)	1.00 (0.99–1.00)	1.00 (0.99–1.00)	1.00 (1.00–1.00)	1.00 (0.99–1.00)	0.98 (0.97–0.99)	0.98 (0.97–0.99)	1.00 (1.00–1.00)
JLCA	0.90 (0.87–0.93)	0.69 (0.59–0.77)	0.80 (0.73–0.85)	1.00 (1.00–1.00)	0.91 (0.85–0.95)	0.71 (0.55–0.82)	0.84 (0.74–0.90)	1.00 (1.00–1.00)	0.90 (0.85–0.93)	0.67 (0.54–0.78)	0.77 (0.67–0.84)	1.00 (1.00–1.00)
MPTA	0.99 (0.98–0.99)	0.95 (0.94–0.97)	0.96 (0.94–0,97)	1.00 (1.00–1.00)	0.98 (0.95–0.99)	0.92 (0.87–0.95)	0.93 (0.88–0.95)	1.00 (1.00–1.00)	0.96 (0.94–0.97)	0.89 (0.83–0.93)	0.89 (0.83–0.92)	1.00 (1.00–1.00)
mLDFA	0.97 (0.97–0.98)	0.90 (0.86–0.93)	0.96 (0.95–0.97)	1.00 (1.00–1.00)	0.98 (0.96–0.99)	0.90 (0.84–0.94)	0.96 (0.93–0.98)	1.00 (1.00–1.00)	0.98 (0.96–0.99)	0.90 (0.84–0.93)	0.96 (0.94–0.98)	1.00 (1.00–1.00)

## Discussion

The most important finding of the present study was that the AI-aided software for automated long-leg alignment measurements produced reliable results for varus malaligned knees pre- and postoperatively after high tibial osteotomy. Although manual readers ranked higher than the AI-driven software, the discrepancies were below 0.5°, which are minor and would not alter clinical decision making.

A detailed deformity analysis to identify varus malalignment is obligatory in patients with medial knee osteoarthritis or patients that require cartilage or medial meniscal repair. When malalignment is addressed concomitantly clinical outcomes after cartilage repair and meniscal repair improve significantly [1]. Analysis of the bony geometry in the coronal plane is usually performed on weight-bearing anteroposterior long-leg radiographs. Manual measurements are time-consuming and can show high inter- and intrareader variability, depending on the experience and fatigue of the observer [3, 25]. Agreement is excellent for HKA and MAD, indicated by an intraclass correlation coefficient of over 0.9 [4]. However, agreement for mLDFA, MPTA and JLCA is reported to be worse [3].

AI can support radiologists and orthopedic surgeons in analyzing radiographs automatically. The literature reports that various AI models have been introduced and tested. Due to the heterogeneity of algorithms applied, varying experience and background of clinical readers as well as different data sets used for performance testing, comparability between studies is limited. However, AI-based analyses offer several advantages, including improved accuracy, time savings, reproducibility and objectivity [22]. However, concerns regarding the application of AI for analyses of radiographs need to be recognized. These include limited transparency and interpretability of AI models, where the internal decision-making process might not be easily understandable. Second, AI algorithms require large amounts of high-quality training data [5]. If these data are biased or unrepresentative for a certain group of patients, it can lead to poor performance of AI models. Hence, it is important to address issues related to data bias to ensure equity and avoid under-representation of certain demographics [6]. Furthermore, effective integration of AI software into the clinical workflow is crucial. For the practical use and application of these models, seamless integration into existing systems (e.g., picture archiving and communication systems) is of utmost importance.

The evidence for AI-aided analyses of long-leg radiographs is rapidly growing. Different study groups developed and validated various AI algorithms for automated measurements on LLRs [13, 19, 23].

The software used in this study (LAMA™) is a commercially available FDA- and CE-marked software that has been studied for native LLRs and implant alignment measurement after TKA [21, 23]. Simon et al. performed a retrospective single-center analysis of 295 native LLRs, where they compared AI measurements with manual readers that constituted a “ground truth” [23]. AI produced an overall accuracy of 89.2% compared with the manual measurements after exclusion of radiographs with metalwork and postoperative images. The mean absolute-deviation for angles was 0.39°–2.19°and 1.45–5.00 mm for length measurements. The intra-class-coefficient (ICC) showed good reliability in all lengths and angles according to Koo et al. (ICC ≥ 0.87). The equivalence-index (*γ*) was between 0.54 and 3.03° for angles and − 0.70 to 1.95 mm for lengths.

The evaluation of LLRs by LAMA™ after TKA showed a high reproducibility and reliability [21]. Correct detection of femoral and tibial components was achieved in 92.1%. Nevertheless, performance was altered in cases of constrained implants, where landmark setting failed in 12.5%. Furthermore, Huber et al. used LAMA™ analyses of preoperative LLRs in osteoarthritic knees prior to TKA to perform functional phenotype and coronal plane alignment of the knee classifications [11]. The authors found gender-specific differences with significant differences between men and women for all radiographic parameters.

However, to our best knowledge this is the first study investigating the performance of an AI application for varus malalignment and after HTO. Especially in the case of osteotomies and joint preserving surgeries a thorough analysis of the leg alignment and detection of the bony deformities is indispensable. The measurements are usually performed manually on long-leg radiographs using a medical imaging software. Still, manual landmark setting showed high intra- and inter-reader variability and poor reproducibility [3, 10, 20]. Furthermore, studies demonstrated that the reliability is affected by the experience of the observers [25].

Our results show mean absolute differences between LAMA™ and mean manual observer measurements of 0.5° or lower for all measurements. Although SUCRA plots show low probabilities that the AI software ranks better than manual readers, except for the AMA, there were no statistically significant differences between manual measurements and the AI-based analyses, neither in native radiographs before surgery nor after osteotomy. The detected differences between the AI and manual measurements were minor and would not influence the clinical decision making-process. The advantage of the AI analysis is the immediate availability of measurement data and detailed information about the leg alignment. The data are automatically evaluated in less than half a minute and immediately available to the treating physician. This instant information together with the reported accuracy and reproducibility could offer advantages in clinical practice, especially in determining the indication for osteotomy and controlling the correction after HTO. The potential clinical relevance includes higher reproducibility, irrespective of the observer’s experience level or fatigue and improved and prompt visualization for patients. The graphical report provided by the AI software highlights important findings and can be used for patient information and education. Furthermore, using the AI-based software leg alignment measurements could easily be available for every long-leg radiograph without the need for additional personnel resources. Hence, even patients with mild varus alignment might be identified faster and directed towards potential joint-preserving therapy.

Previous studies have demonstrated high accuracy of AI-aided analyses in native LLRs for leg length and alignment [13, 23]. However, this is the first study investigating an AI application for automated measurements specifically in a population of patients with varus malalignment that underwent HTO. Our results confirm the feasibility of fully automated measurements and the high accuracy in native radiographs. In the preoperative measurements differences between LAMA™ and manual observers were as low as 0.21° or lower. Despite our findings indicating a low likelihood of the AI outperforming human readers in terms of absolute deviation from the median, the impact of these deviations on clinical decision-making would be minimal. Consequently, the application for long-leg analysis in patients with varus leg alignment is feasible for clinical practice. Our findings confirm a considerable intra-reader variability for manual measurements that has been reported in the literature. While HKA and MAD showed intra-observer ICCs of 0.98 or higher, repeated measurements for mLDFA and MPTA showed a higher variability. In accordance to previous studies, JLCA showed by far the highest variability with ICCs demonstrating moderate to excellent reliability. In contrast, repeated measurements for AI-aided measurements demonstrated perfect reproducibility with an ICC of 1.0 for all measurements preoperatively and after HTO. These excellent results for measurements postoperatively after HTO showed no significant differences compared to manual measurements. The analysis of postoperative X-rays after osteotomy might pose a challenge to the software due to altered bone morphology and the presence of osteosynthesis material. In our study, the AI software showed erroneous results in 9/1140 measurements (0.79%). These incorrect measurements were found on two native preoperative and three postoperative radiographs. The detailed error analysis revealed that an incorrect landmark placement was the primary cause for erroneous results, both pre- and postoperatively. In two of the postoperative cases, the incorrect landmark setting occurred in the proximal tibia, where the proximal tibial joint line was placed at the plate medially rather than the tibial plateau. Naturally, this resulted in significant deviations for JLCA and MPTA. In the third postoperative erroneous case the distal femoral joint line was incorrectly marked and JLCA and mLDFA showed implausible values.

Preoperatively, in one case the distal femoral joint line was incorrectly marked due to advanced medial OA, resulting in significant deviations for JLCA and mLDFA. In the second erroneous preoperative image, the calibration ball was incorrectly identified resulting in an erroneous estimation of the magnification factor and incorrect MAD values, while angle measurements were not altered. It needs to be emphasized that all results provided by an AI software in the medical field need to be confirmed by medical professionals. The erroneous measurements in this study are illustrated in the supplement material and were clearly discernible for physicians with experience in musculoskeletal imaging.

This study has some limitations that need to be considered when interpreting the results. First, sample size was relatively small, which may limit the generalizability of the findings in larger populations. Second, the accuracy of the automated analysis is dependent on the quality of the radiographs, and variations in image quality across different healthcare facilities may affect the algorithm’s performance. Furthermore, the study compared the automated analysis with manual measurements that are not infallible and may themselves have inherent limitations.

However, the findings of this study support the use of AI-based analyses for long-leg radiographs in patients with varus malalignment and after HTO. In clinical practice, the use of an AI-based software that offers fully automated measurements could increase the availability of accurate alignment measurements without the need for additional personnel resources. An instant visual report with accurate results accompanying each image could enhance awareness and enable early detection of malalignment. This study demonstrates that the AI-aided software is also applicable for postoperative radiographs following HTO. This enables an accurate analysis of postoperative alignment and can be used for patient education. Furthermore, this technology offers the potential to enhance the precision and reproducibility, mitigating the significant deviations in manual measurements.

## Conclusion

An AI-based software can provide accurate and reproducible measurements of varus malalignment and postoperatively after HTO. Consequently, the implementation for fully automated long-leg analyses is feasible in the clinical setting for patients with varus leg alignment and after HTO and could support clinical workflows. However, results need to be validated by medical professionals.

### Supplementary Information

Below is the link to the electronic supplementary material.

Supplementary file1 (DOCX 32365 KB)

## Data Availability

Not applicable.

## Abbreviations

ACI: Autologous chondrocyte implantation
AI: Artificial intelligence
AMA: Anatomical–mechanical axis angle
CNN: Convolutional deep neural network
DICOM: Digital Imaging and Communications in Medicine
HKA: Hip–knee–ankle angle
HTO: High tibial osteotomy
ICC: Intra-class-coefficient
JLCA: Joint line convergence angle
LAMA™: Leg Angle Measurement Assistant
LLR: Long leg radiograph
MAD: Mechanical axis deviation
mLDFA: Mechanical lateral distal femoral angle
mLDTA: Mechanical lateral distal tibial angle
mLPFA: Mechanical lateral proximal femoral angle
MPTA: Medial proximal tibial angle
OA: Osteoarthritis
SUCRA: Surface under the cumulative ranking
TKA: Total knee arthroplasty
