# Supplementary material for: Artificial intelligence-based analyses of varus leg alignment and after high tibial osteotomy show high accuracy and reproducibility
Source: Knee Surg Sports Traumatol Arthrosc. 2023 Nov 17;31(12):5885–95. doi: 10.1007/s00167-023-07644-0 (PMC10719140; doi:10.1007/s00167-023-07644-0)
Supplement: Supplementary file 1 — Supplementary file1 (DOCX 32365 KB) [file 167_2023_7644_MOESM1_ESM.docx]

Supplement 1

|  | **Pre-op (LAMA™)** | **Pre-op (manual)** | **p-value** | **Post-op (LAMA™)** | **Post-op (manual)** | **p-value** |
| --- | --- | --- | --- | --- | --- | --- |
| **HKA (°)** | 5.36 (±3.03) | 5.47 (±2.90) | 0.7985 | -0.70 (±2.34) | -0.54 (±2.31) | 0.6359 |
| **MAD (mm)** | 19.38 (±11.39) | 20.17 (±10.99) | 0.6272 | -2.68 (±8.75) | -2.10 (±8.61) | 0.6457 |
| **JLCA (°)** | 2.23 (±2.0) | 2.03 (±1.84) | 0.4741 | 1.37 (±2.86) | 1.78 (±1.83) | 0.2407 |
| **MPTA (°)** | 86.29 (±2.42) | 86.08 (±2.34) | 0.2100 | 91.6 (±3.0) | 91.81 (±2.54) | 0.6032 |
| **mLDFA (°)** | 89.42 (±1.99) | 89.52 (±5.3) | 0.8500 | 89.48 (±2.96) | 89.49 (±2.19) | 0.9789 |

Table 1: Preoperative and postoperative radiographic measurements for LAMA™ and mean manual measurements. All values are reported as mean ± standard deviation (SD). For MAD and HKA positive values (+) indicate varus, while negative values (-) indicate


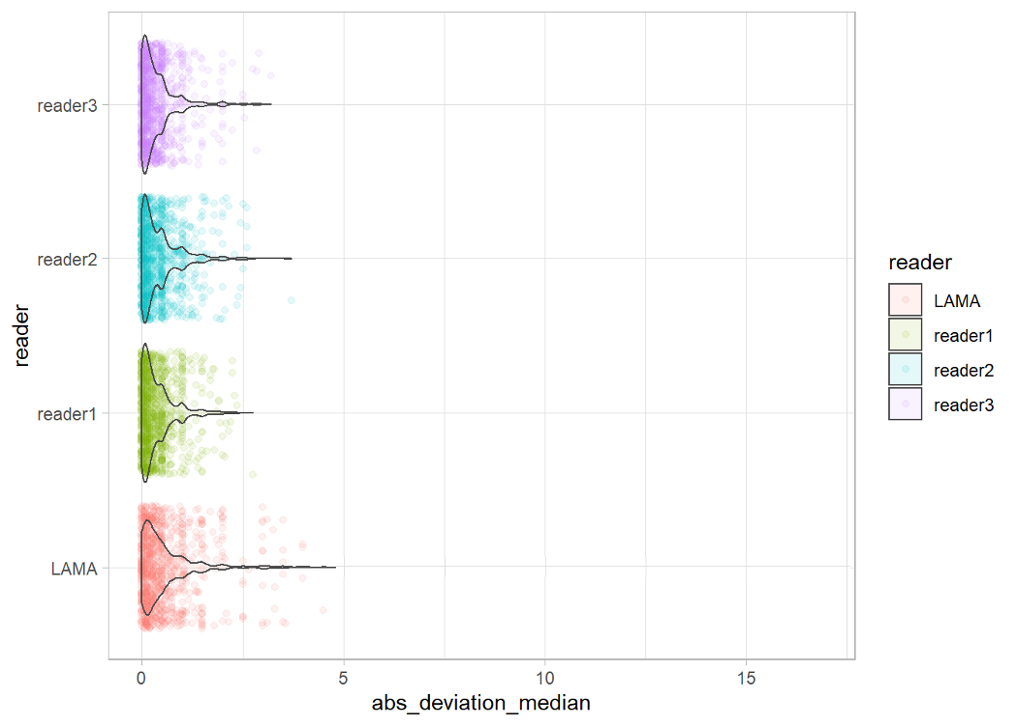


Figure 1: Absolute deviation from the median for each individual observation for all readers and LAMA™.


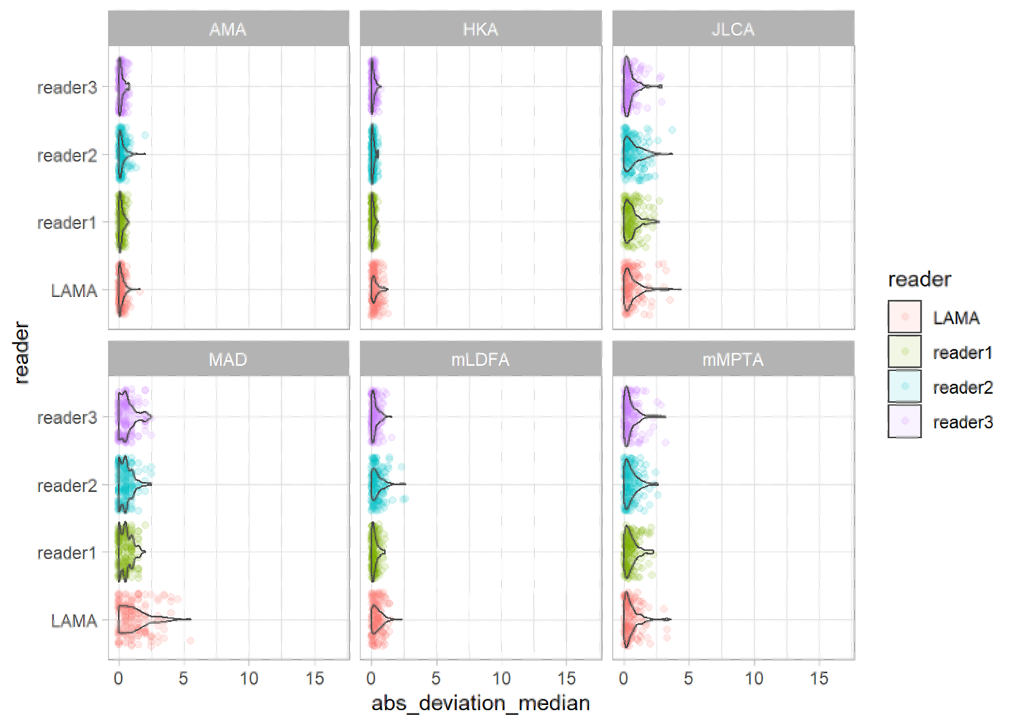


Figure 2: Absolute deviations from the median for each individual observation for all measurements for reader 1-3 and LAMA™.


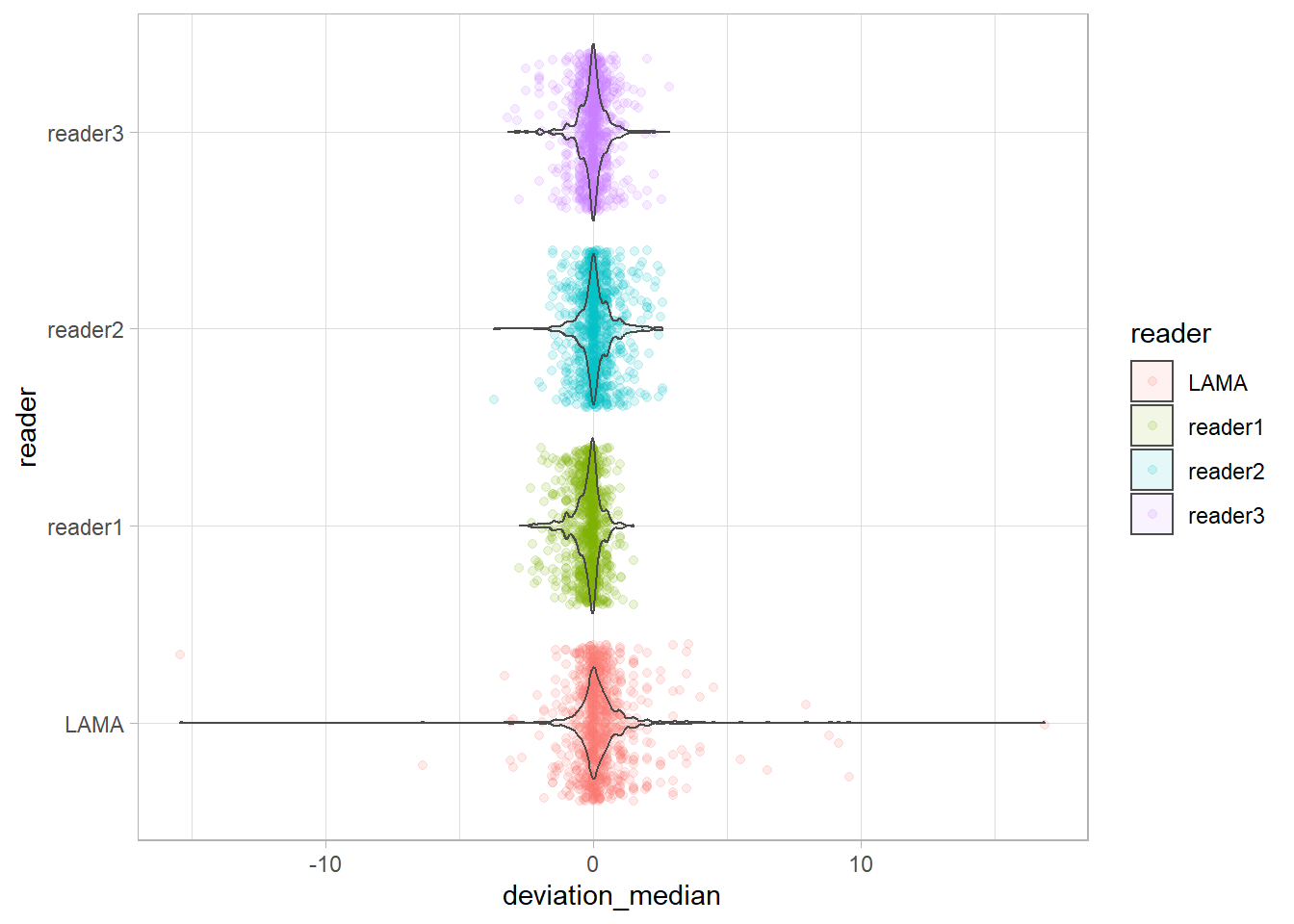


Figure 3: Deviation from the median for each individual observation of all readers and LAMA™ including outlier measurements


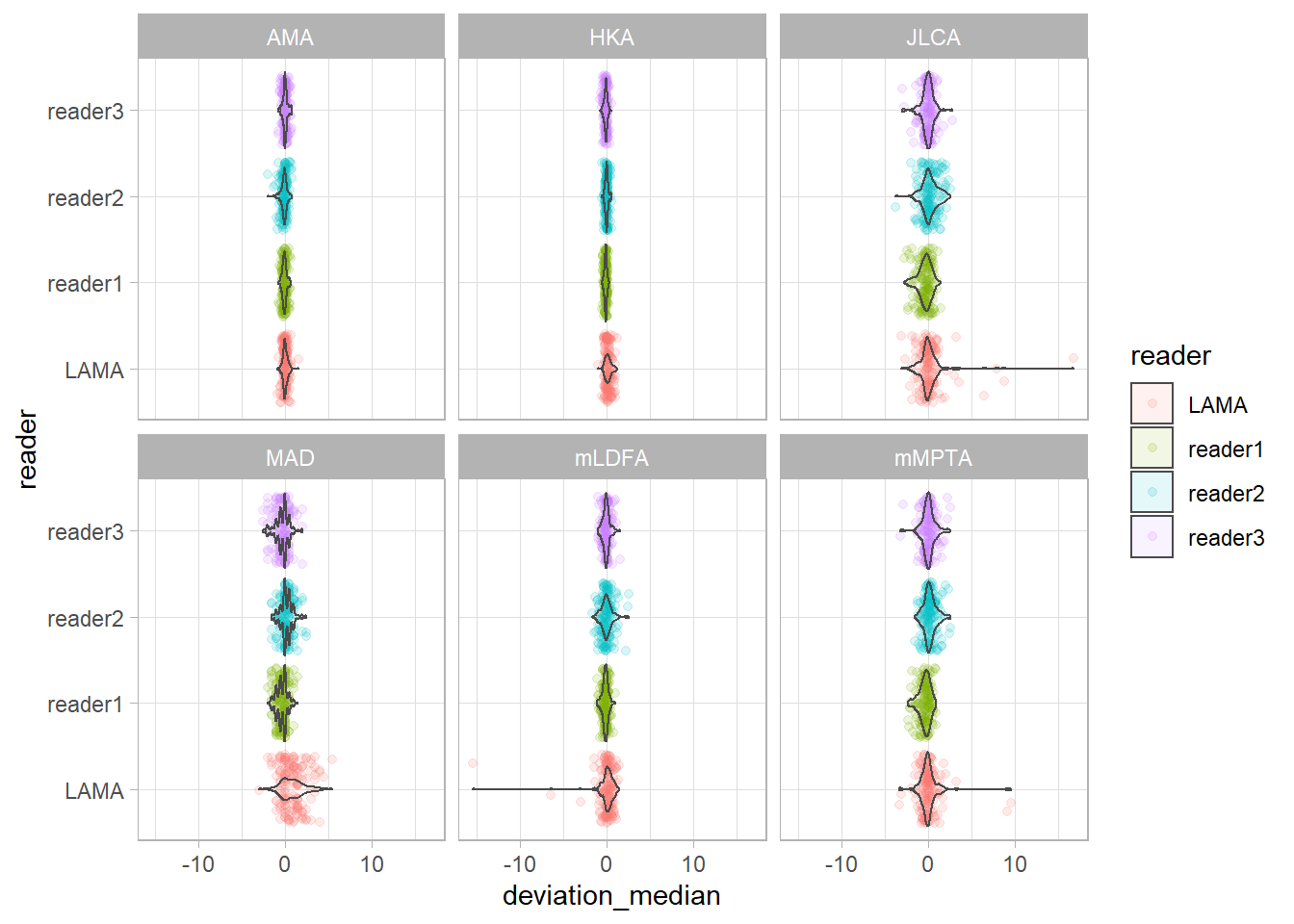


Figure 4: Deviations from the median for each individual observation for all measurements for reader 1-3 and LAMA™ including outlier measurements


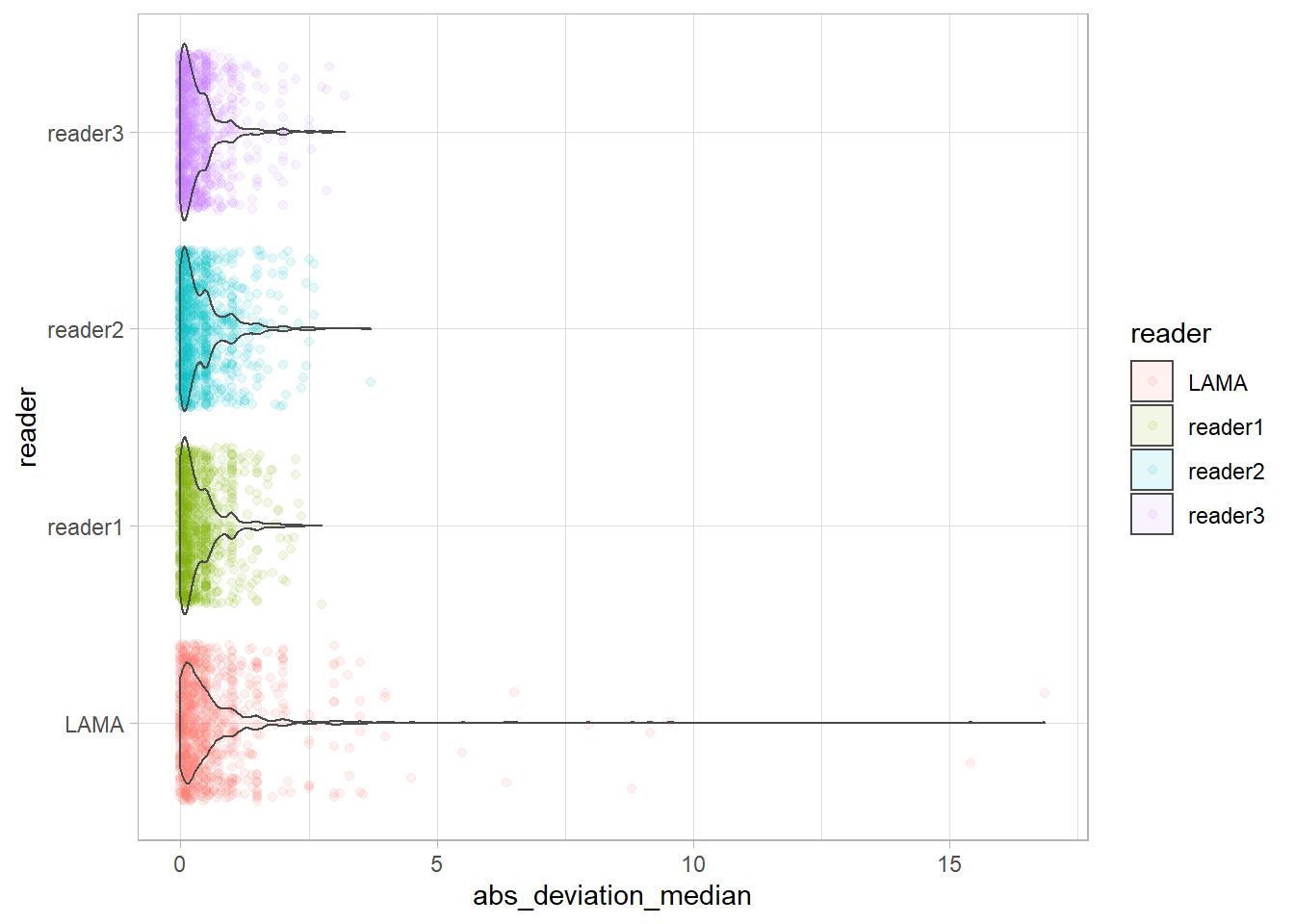


Figure 5: Absolute deviation from the median for each individual observation for all readers and LAMA™ including outlier measurements


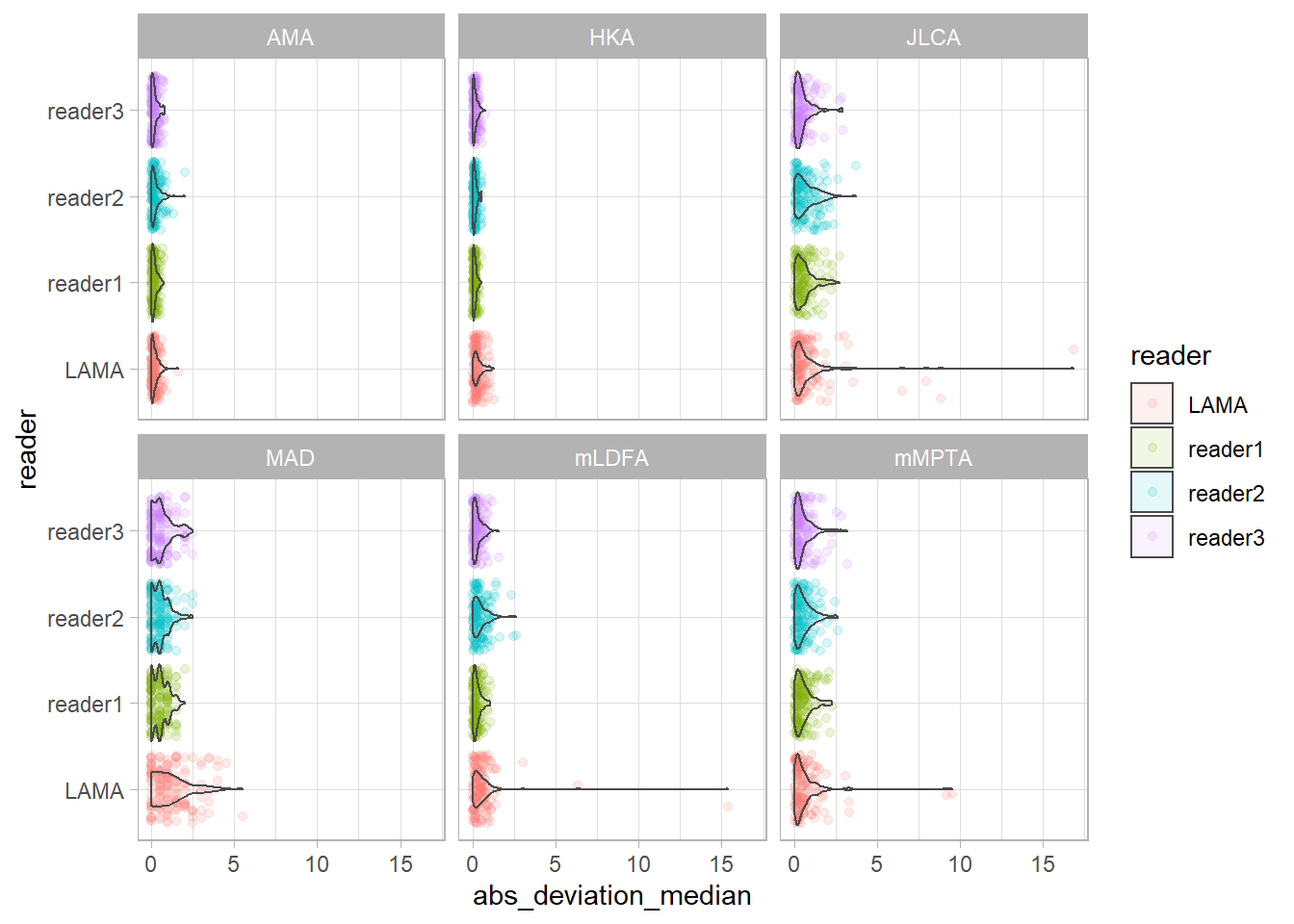


Figure 6: Absolute deviations from the median for each individual observation for all measurements for reader 1-3 and LAMA™ including outlier measurements


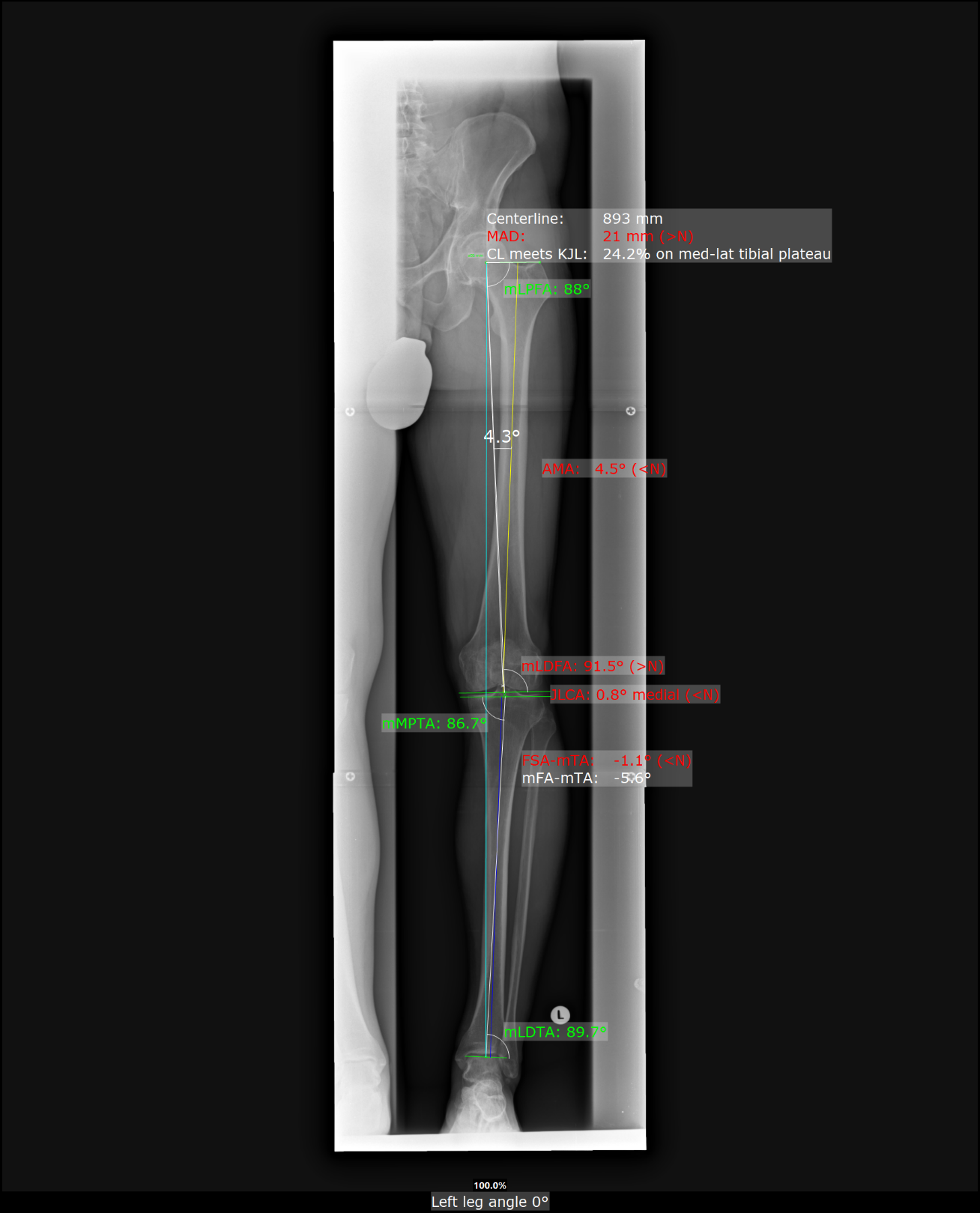

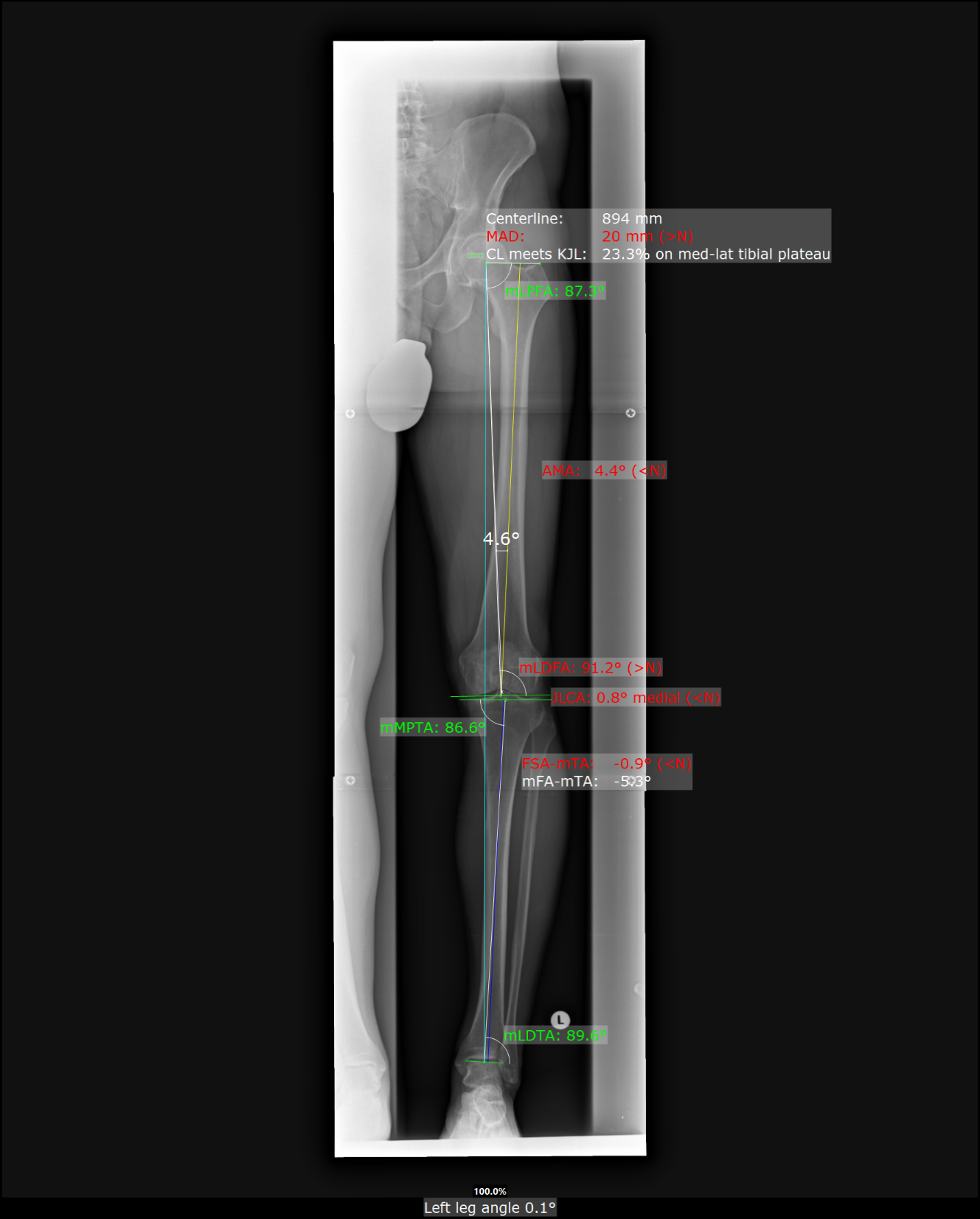


Figure 7: Erroneous AI measurement in a native long-leg radiograph with incorrectly identified calibration ball resulting in incorrect MAD result.


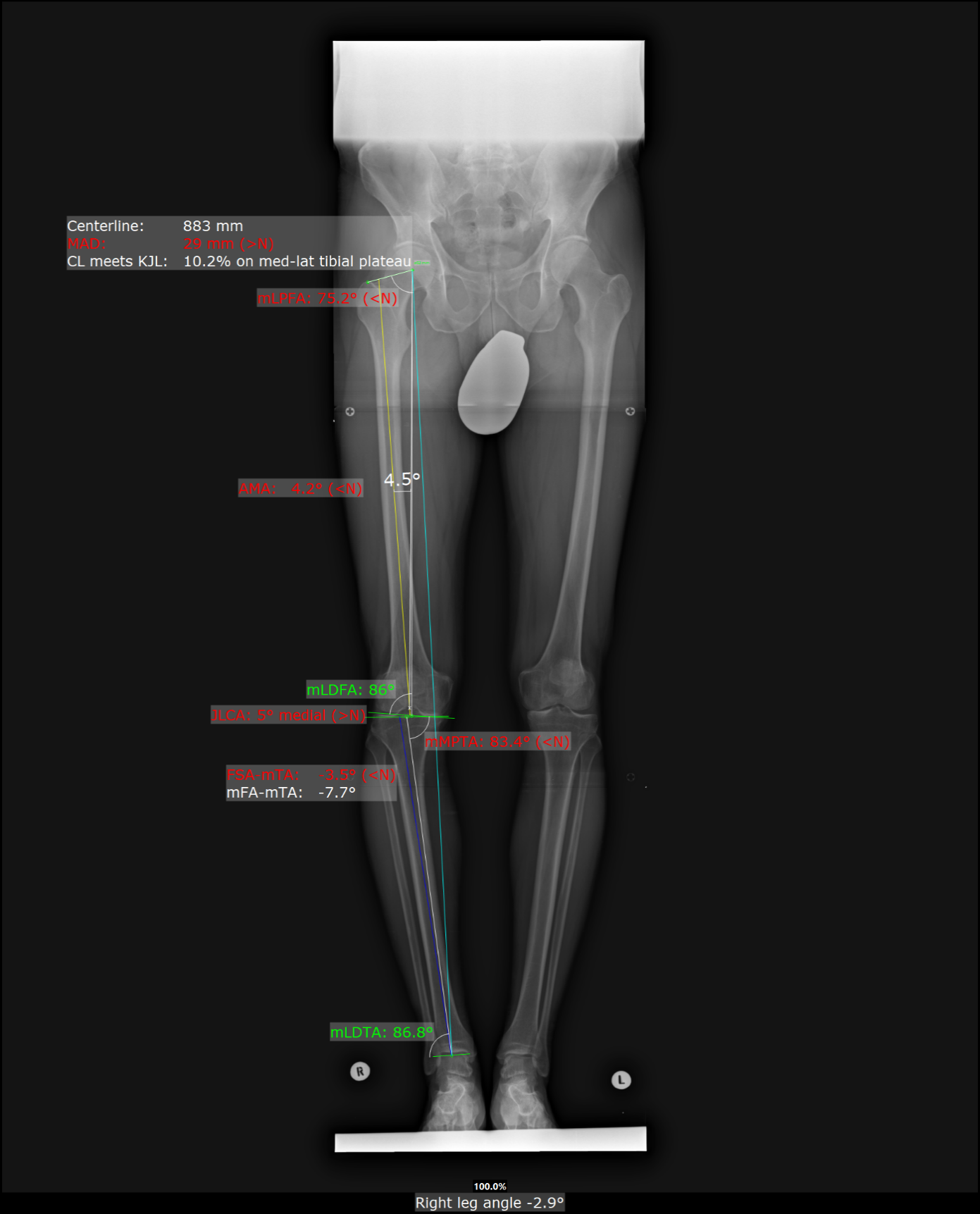

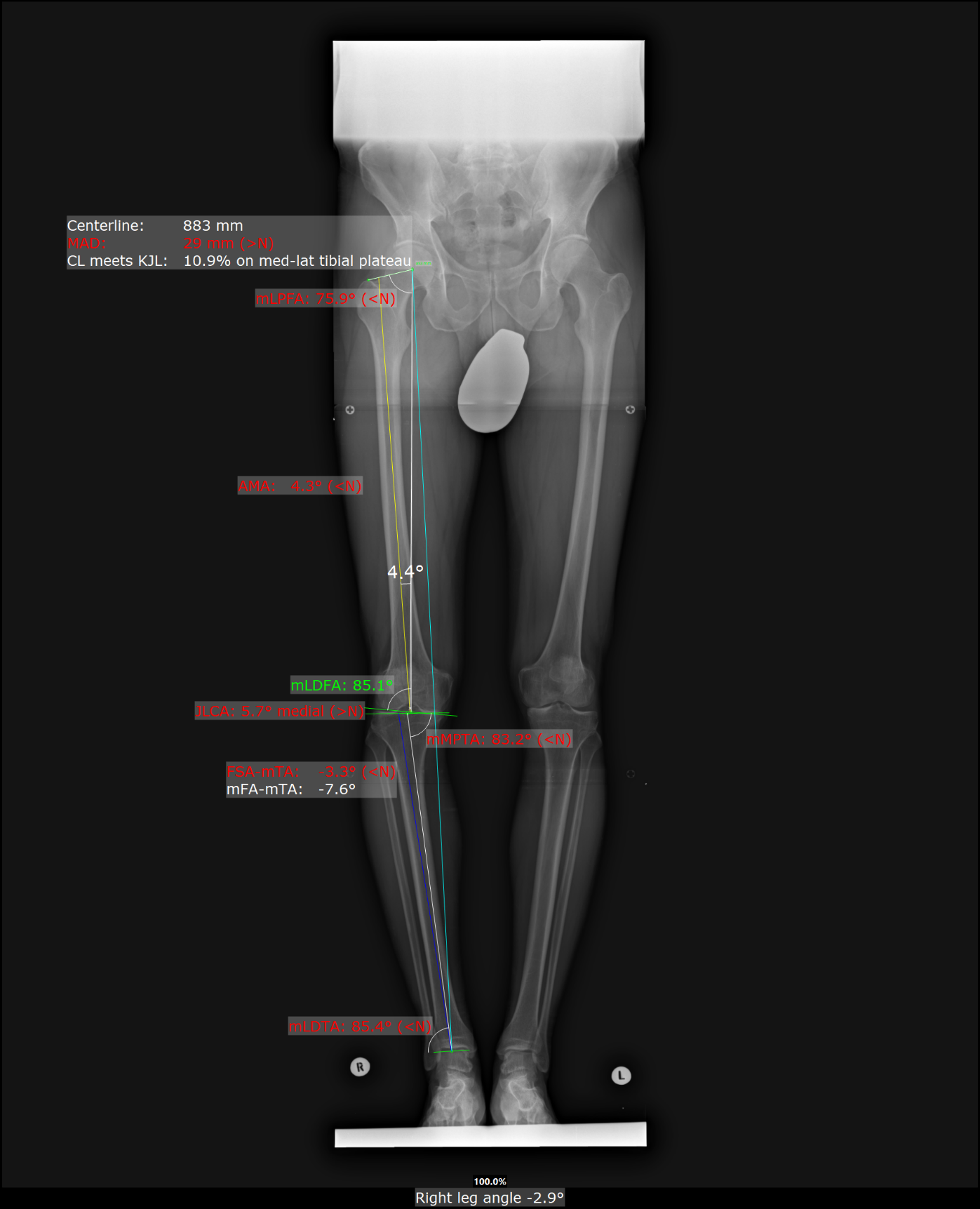


Figure 8: Erroneous AI measurement in a native long-leg radiograph with incorrect identification of the distal femoral joint line and incorrect JLCA and mLDFA results.


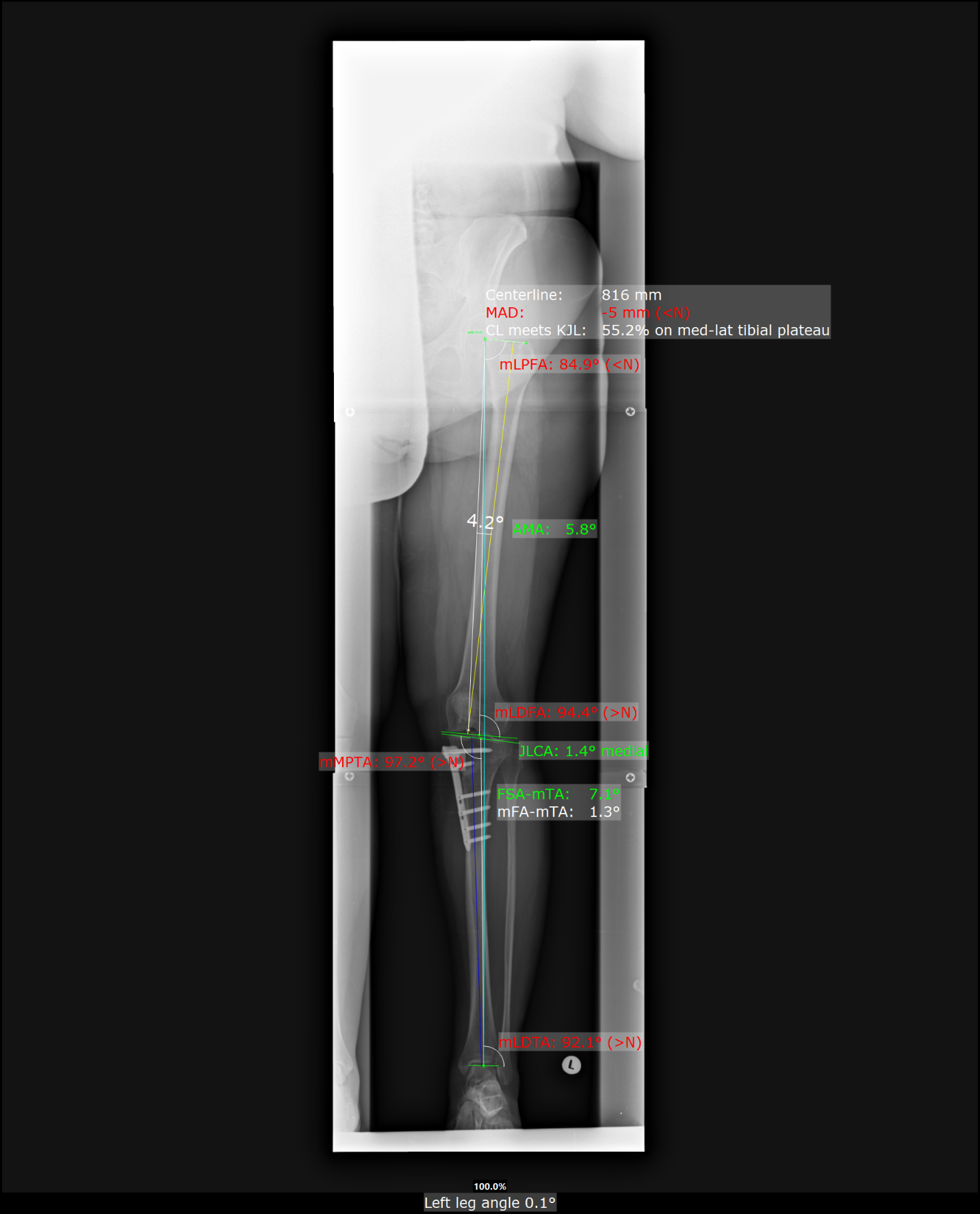

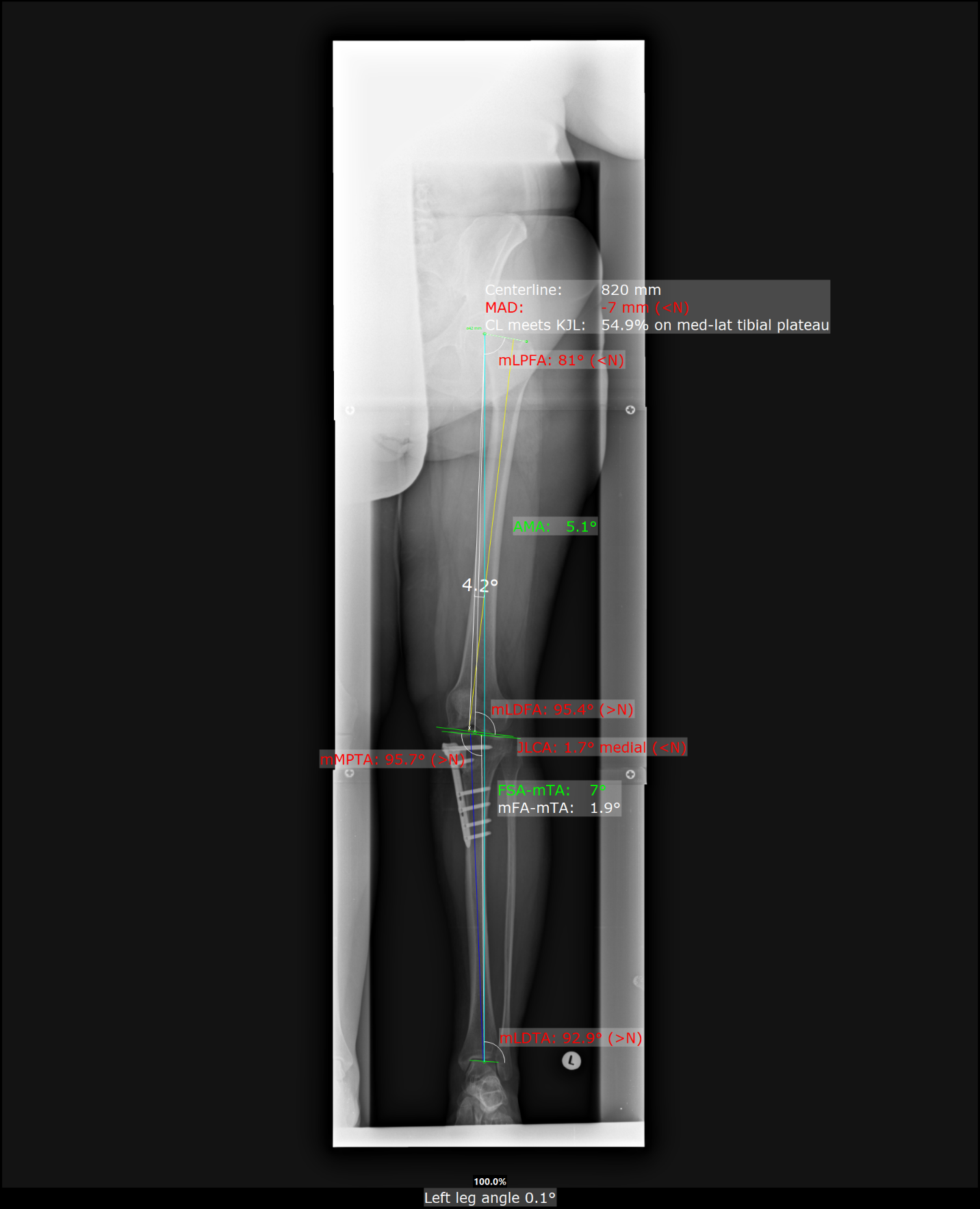


Figure 9: Erroneous AI measurement in a postoperative long-leg radiograph after HTO with incorrect identification of the distal femoral joint line resulting in incorrect JLCA and mLDFA results.


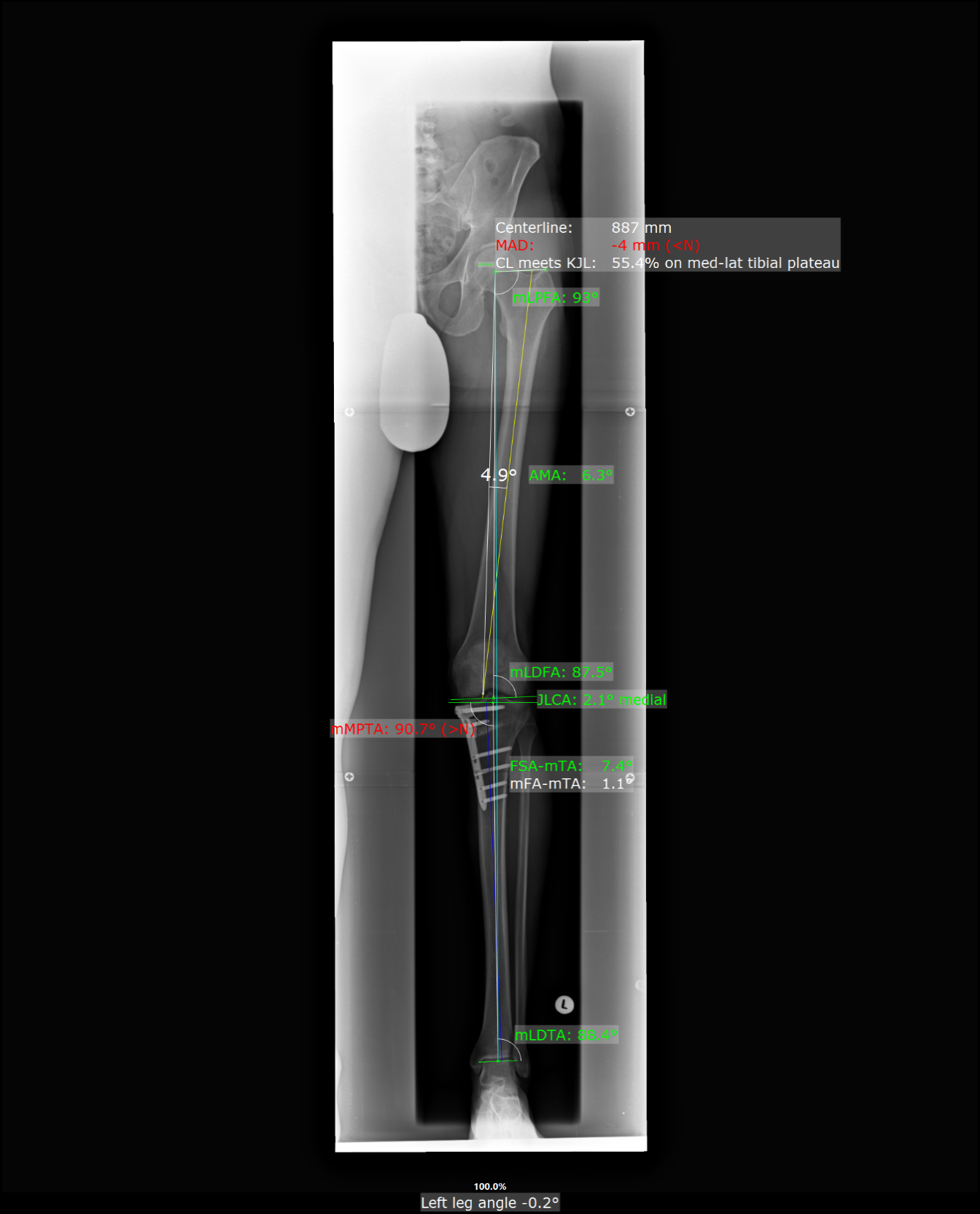

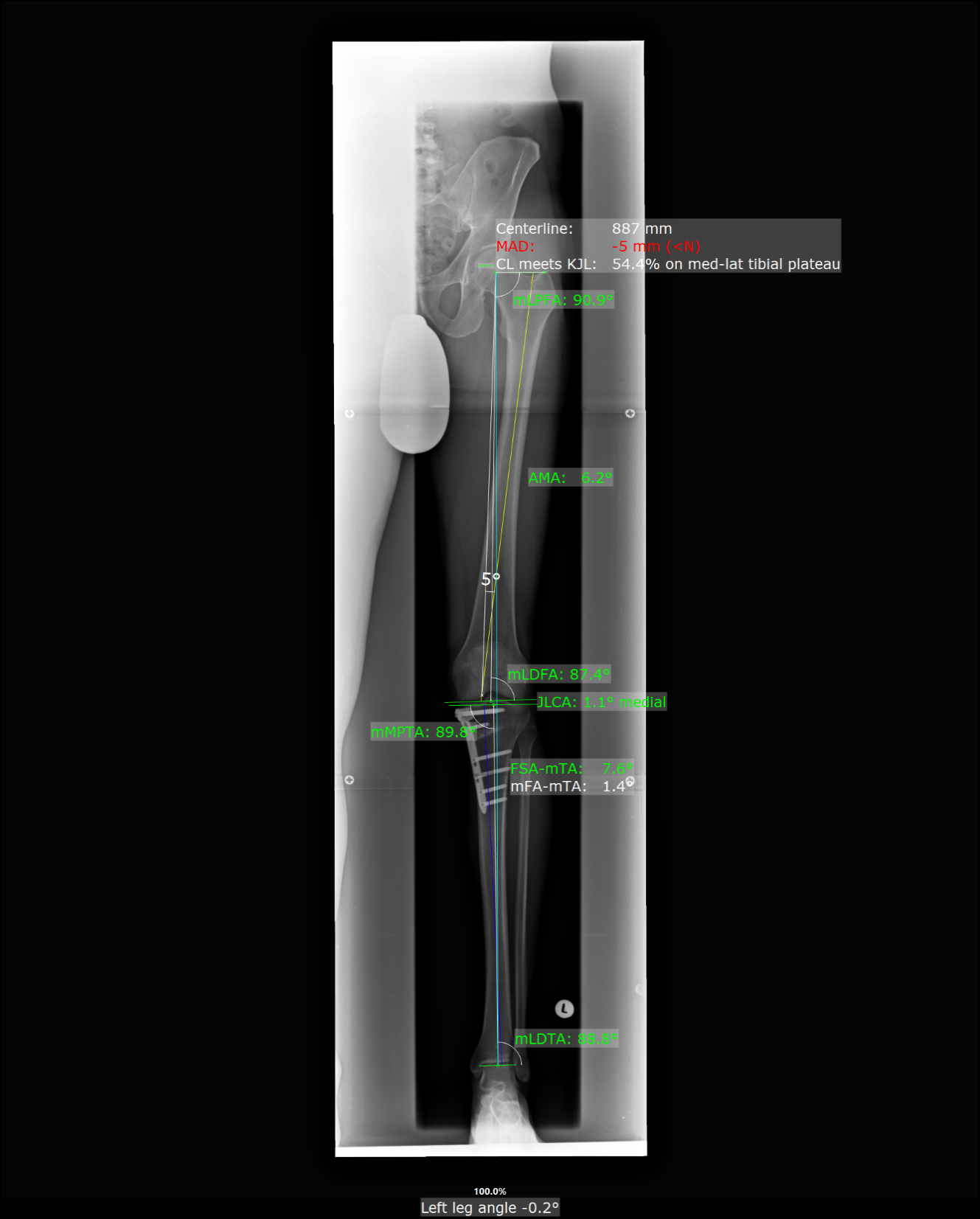


Figure 10: Erroneous AI measurement in postoperative long-leg radiograph after HTO with incorrect identification of the proximal tibial joint line resulting in incorrect JLCA and MPTA results.


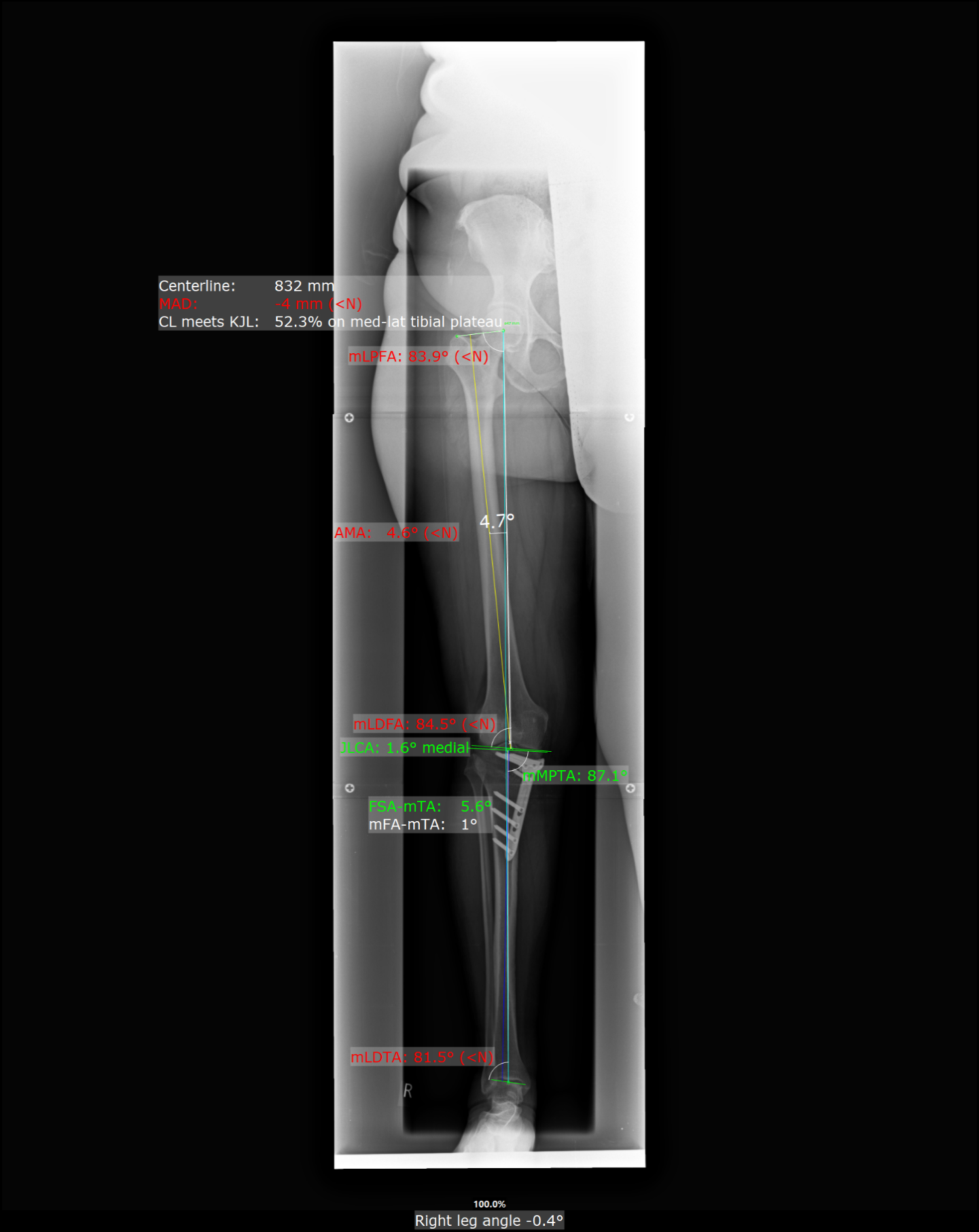

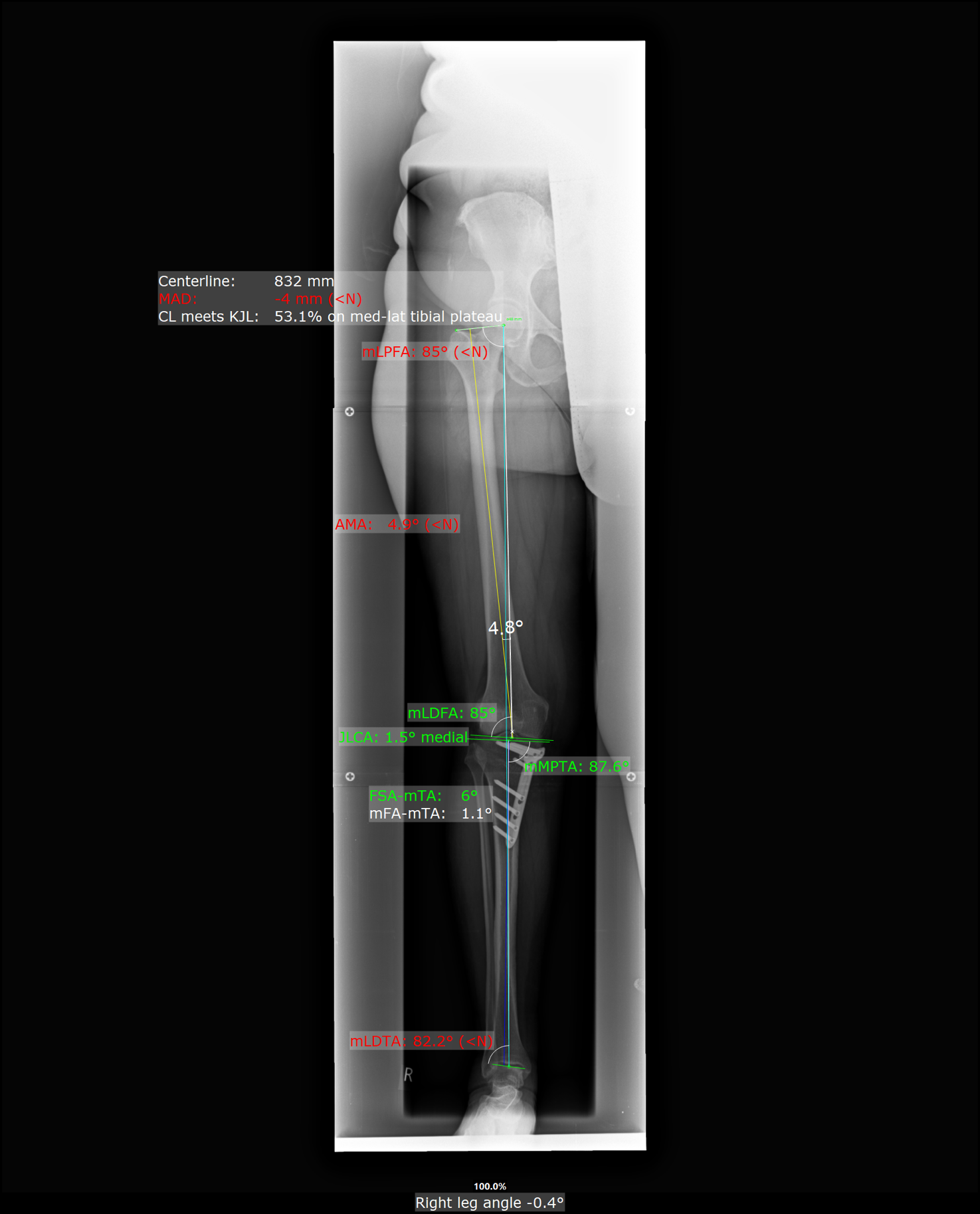


Figure 11: Erroneous AI measurement in postoperative long-leg radiograph after HTO with incorrect identification of the proximal tibial joint line resulting in incorrect JLCA and MPTA results.
